# Emotional, Relational, and Identity Shifts in Aphasia Caregiving: An Interpretative Phenomenological Analysis of Care Partner Experiences

**DOI:** 10.1111/1460-6984.70290

**Published:** 2026-07-10

**Authors:** Eleanor Siegle, Brooke Boxrud, Stewart A. Shankman, Claudia M. Haase, Sameer A. Ashaie

**Affiliations:** ^1^ Think and Speak Lab Shirley Ryan AbilityLab Chicago Illinois USA; ^2^ Stephen M. Stahl Center for Psychiatric Neuroscience Department of Psychiatry and Behavioral Sciences Feinberg School of Medicine Northwestern University Chicago Illinois USA; ^3^ School of Education and Social Policy Northwestern University Evanston Illinois USA; ^4^ Roxelyn and Richard Pepper Department of Communication Sciences and Disorders Northwestern University Evanston Illinois USA; ^5^ Department of Physical Medicine and Rehabilitation Feinberg School of Medicine Northwestern University Chicago Illinois USA

**Keywords:** aphasia, care partner, caregiver, emotional labour, identity, IPA

## Abstract

**Background:**

Care partners of people with aphasia, an acquired language disorder affecting multiple linguistic modalities, face significant life changes when assuming caregiving responsibilities. These changes affect both physical and psychological well‐being, yet the nuanced experiences of care partners remain underexplored.

**Aims:**

Our aim was to explore the subjective experiences of informal care partners of people with aphasia and understand how caregiving influences their identities, relationships and emotional well‐being.

**Methods & Procedures:**

Thirteen informal care partners participated in three focus groups (4–5 care partners per group) using a semi‐structured interview format. Data were analysed using interpretative phenomenological analysis (IPA). IPA is a qualitative approach grounded in a double hermeneutic process in which participants make sense of their experiences and researchers engage with and interpret that sense‐making to support a richer analysis than surface‐level description could provide.

**Outcomes & Results:**

Analysis revealed four superordinate themes: (1) Shapeshifting to manage many roles; (2) loss of intimacy; (3) relentless positivity and (4) resentment, anger and sadness.

**Conclusions & Implications:**

Caregiving in aphasia involves identity transformations, emotional labour and relational strain. Findings highlight the need for interventions that address care partners’ psychosocial adjustment, normalize negative emotions, and support authentic emotional exchange within aphasia‐caregiving relationships.

**WHAT THIS PAPER ADDS:**

*What is already known on the subject*

- Care partner experiences in aphasia caregiving have been well‐documented, however, current research in the field predominantly relies on descriptive qualitative methods or quantitative burden measures. In comparison, the lived and interpretive dimensions of caregiving in aphasia remain underexplored.

*What this study adds to existing knowledge*

- This study examines the subjective experiences of caregiving in aphasia using interpretative phenomenological analysis, as this method allows for a deeper look at how care partners make sense of the identity, relational, and emotional‐level dimensions of caregiving.

*What are the clinical implications of this study?*

- To better support care partners. overall well‐being, clinicians could incorporate these findings into family education that includes facilitated discussions or therapeutic exercises to address topics such as identity shifts and the emotional and relational complexities of caregiving.

## 1 Introduction

Stroke is one of the leading causes of long‐term disability, significantly impacting both stroke survivors and their care partners, typically family members. Recovery from stroke often requires ongoing assistance with daily‐life activities, leading many stroke survivors to rely heavily on their care partners for support (Dewey et al. 2002). While caregiving can foster positive outcomes (e.g. strengthened family bonds, pride, personal meaning), it is frequently associated with negative experiences (e.g. increased stress, burden, loneliness, fatigue and depression) (Berg et al. 2005; Rigby et al. 2009; Chen et al. 2017; Hu et al. 2018; Denham et al. 2022).

These negative impacts may be amplified when caring for individuals with post‐stroke aphasia, an acquired disorder that affects multiple aspects of language (Grawburg et al. 2013; McGurk and Kneebone 2013). Care partners of people with post‐stroke aphasia often report lower levels of social satisfaction and smaller social networks, along with increased financial burden, reduced levels of intimacy, and overall emotional issues compared to care partners of people without post‐stroke aphasia (Bakas et al. 2006; Le Dorze and Signori 2010; Stead and White 2019; Haley et al. 2020; Aslan and Altuntaş 2024). Despite taking on new responsibilities and adopting communication strategies to better support people with aphasia, the psychosocial needs of care partners are often overlooked (Simmons‐Mackie and Cherney 2018; Stead and White 2019). Furthermore, a lack of post‐discharge information from healthcare professionals can further exacerbate these challenges and increase care partner burden (Le Dorze and Signori 2010). These challenges can contribute to ‘third‐party disability’, in which care partners’ own health and functioning are affected by the demands of caregiving, extending the impact of the original disability beyond the affected individual to the entire family system (Grawburg et al. 2019). Although these challenges exist, there is limited research on how care partners interpret and make sense of their caregiving experiences, as well as how they navigate the complex emotional, relational and identity‐related dimensions of caregiving.

According to caregiver identity theory (CIT), caregiving is a dynamic process that disrupts pre‐existing familial roles (e.g. sibling, spouse) and forces family members to adopt new identities dominated by their roles as care partners (Montgomery and Kosloski 2009). The disparity between the practical demands of caregiving and a caregiver's personal identity is, according to CIT, a significant cause of burden and stress (Montgomery and Kosloski 2009; Montgomery et al. 2011). As caregivers take on responsibilities that diverge from their prior relational identity, they may experience a sense of loss or conflict between their former selves and their new identities as caregivers (Ribeiro et al. 2021).

CIT is an especially relevant theory for understanding caregiving in the context of post‐stroke aphasia, where communication changes can profoundly alter relationships and shared meaning‐making (Harcourt et al. 2025). Since the onset of post‐stroke aphasia is sudden, this change in relational identity is abrupt and may not give care partners enough time to negotiate the burden of their new identity. Furthermore, because aphasia reshapes how people connect and communicate, care partners may face additional strain as they work to build a shared relational identity that fits new modes of communication (Iwasaki et al. 2023).

Grounded in CIT, this study used interpretative phenomenological analysis (IPA) to investigate how care partners make sense of their experiences in aphasia caregiving and how emotional and relational dynamics influence their involvement and identity formation. This theoretical (CIT) and methodological (IPA) pairing provided a coherent framework to provide insights that go beyond what traditional burden measures or descriptive qualitative approaches typically capture.

## 2 Materials & Methods

### 2.1 Overview

The phenomenology of IPA centres around the lived experiences of participants and directs analyses toward the meanings they attribute to different contexts (Pietkiewicz and Smith 2014). Data were collected through focus groups, which facilitated both individual meaning‐making and collective understanding, offering insights that may be missed in individual interviews (Kitzinger 1995; Wilkinson 1998). Consistent with IPA's idiographic process, participant engagement guided the analysis before identifying superordinate themes that captured patterns of meaning across groups (Smith 2004; Tomkins and Eatough 2010). Additionally, the double hermeneutic nature of IPA, where participants make sense of their own experiences and the researcher engages with and interprets those understandings, supported a richer analysis than surface‐level description could provide (Jonathan A. Smith et al. 1999; Pietkiewicz and Smith 2014; Smith et al. 2021).

### 2.2 Participants

Secondary data analysis was conducted on data from a focus group study with a total of thirteen care partners of people with aphasia. Care partners were recruited from the community through phone calls and e‐mails. Their ages ranged from 35 to 77 years, and their relationships to the people with aphasia they care for varied (e.g. spouses, parents). Table 1 summarizes the participant characteristics. Demographic information was also collected for the individuals with aphasia whom the care partners supported. All people with aphasia had a left‐hemisphere stroke and no comorbid neurological conditions impacting their communication. Their aphasia severity ranged from severe to mild on the Aphasia Severity Rating Scale (Simmons‐Mackie and Cherney 2018), and time post‐onset ranged from 1 to 17 years (M = 6.27, SD = 5.01). All participants provided written consent prior to beginning the study, and the study was approved by the Institutional Review Board. Pseudonyms are used throughout the manuscript to protect the identities of care partners.

**TABLE 1:** Participant demographic characteristics (*n* = 13).

Variable	M (SD)	*n* (%)
Age (years)	57.08 (12.77)	
Time spent caring (years)	5.78 (4.11)	
Gender		
Women		8 (61.5%)
Men		5 (38.5%)
Race/ethnicity		
White		8 (61.5%)
Black/African American		2 (15.4%)
Asian/Pacific Islander		1 (7.7%)
Prefer not to say		2 (15.4%)
Education		
High school or less		2 (15.4%)
Some college		1 (7.7%)
Trade School		1 (7.7%)
Associate's degree		1 (7.7%)
Bachelor's degree		1 (7.7%)
Graduate degree		7 (53.8%)
Relationship to PWA		
Spouse		9 (69.2%)
Parent		2 (15.4%)
Adult Child		1 (7.7%)
Sibling		1 (7.7%)

Abbreviation: PWA, person with aphasia.

### 2.3 Data Collection

Three focus groups, each containing four to five care partners, were conducted via Zoom and lasted between 2 and 3 h. The focus groups followed a semi‐structured interview format, with questions centered on mental health and aphasia. Care partners were encouraged to expand upon each question during the focus groups by describing their firsthand experiences regarding caregiving. The focus groups were facilitated by a speech‐language pathologist with co‐facilitation provided by one of the first authors. The recordings were saved on Zoom, and the automatic transcripts were manually corrected line‐by‐line by the first author to ensure accuracy. The corrected transcripts were de‐identified and imported into Dedoose 9.2 for qualitative analysis.

### 2.4 Data Analysis

IPA was used for the qualitative analysis. While IPA is typically used in individual interviews, given its emphasis on capturing a person's subjective lived experience, it also has merit in focus group settings (Tomkins and Eatough 2010; Phillips et al. 2016; Love et al. 2020). Additionally, differing perspectives on the same topic in a focus group can provide a more complex picture of a particular phenomenon than one‐on‐one interviews. In group settings, participants can expand on topics introduced by others, which enhances the depth and richness of the data (Bradbury‐Jones et al. 2009). Moreover, individual accounts are still present in a focus group setting, as participants are able to share stories regarding their individual experiences that reflect the idiographic philosophy of IPA (Bradbury‐Jones et al. 2009; Pietkiewicz and Smith 2014). Care partners in our study shared idiographic accounts of caring for someone with aphasia, while both relating to and diverging from other participants’ experiences. In this way, the group setting doesn't dilute IPA's depth; it strengthens it by making visible the ways care partners collectively interpret their experiences alongside their individual accounts.

One of the first authors followed the established steps for IPA and ensured rigour, trustworthiness, and reflexivity throughout the analytic process (De Witt and Ploeg 2006; Rodham et al. 2015). First, she immersed herself in the data by taking notes during the focus groups, re‐watching the focus group videos and recording initial observations. By engaging closely with the raw audio and video data and not solely relying on transcripts, she reduced the risk of imposing preconceptions and potential biases onto participants’ accounts (Rodham et al. 2015). She used the code function in the Dedoose software to track the development of potential themes from each of the focus group transcripts, noting observations specifically related to the impact of aphasia on care partners. She maintained reflexive notes, continually checking her assumptions, positionality and interpretations against the data. She met with the last author to discuss the initial findings and potential superordinate themes. Following this, she identified which potential themes stood out in relation to the research topic and organized them into clusters. She then discussed these thematic clusters with the last author and generated superordinate themes from the data that encapsulated the experience of the impact of aphasia on care partners. Finally, to preserve the idiographic accounts of participants, she made another pass through the data, focusing on each individual one at a time, making note of where individuals’ accounts aligned with each theme and checking whether participants gave data that corresponded to the developed themes (Love et al. 2020). Additionally, the other first author made an independent pass through the data to verify alignment with the first author's interpretations and to support analytic consensus. The analytic steps are outlined in Table 2 (Jonathan A. Smith et al. 1999; Tierney‐Hendricks et al. 2023). Appendix 1 contains the steps of the idiographic loop (Step 6 of IPA analysis).

**TABLE 2:** The interpretative phenomenological analysis (IPA) approach.

Step	Overview	Description
1	Immersion in the data	Repeatedly listening to, watching, or reading the content, taking notes, and creating comments/reflecting on content in memos
2	Initial coding	Creating codes to document the initial analysis of each transcript, with several coding fragments created
3	Initial development of themes	Developing themes from codes
4	Looking for connections among the themes	Clustering the developed themes to form superordinate themes and relating them back to the transcripts
5	Repeat Steps 1–4 for each transcript	
6	Idiographic loop + looking for themes across all participants	Reviewing each transcript to verify that themes align with each member of the focus groups, documenting these in a table to observe theme frequency across participants, and determining which were most salient

## 3 Results

Four superordinate themes and nine subthemes were developed that capture the complex and identity‐impacting dimensions of caregiving for care partners of people with aphasia. See Table 3 for an overview of the results.

**TABLE 3:** Results overview.

Theme	Subthemes	Example Quotes
1. Shapeshifting to manage many roles	1.1 Beyond the sole provider 1.2 An informal therapist 1.3 A social middleman	*He was… the person who did all of the finances… I learned as we went*. *My mom was like, ‘Hey, did you watch this show?’ I'm like ok, tell me about it… even if I've seen it already…* *I had to explain to family members… he's just trying. He just wants to talk to somebody…*
2. Loss of intimacy	2.1 Communication without depth 2.2 Pretending in conversations 2.3 Erosion of intimacy	*…there's no depth, you know… communication, but no depth…* *I keep saying I understand it… by now my mind has lost complete interest in whatever we were talking about*. *The intimacy… You know, the int‐ intimacy definitely changed…*
3. Relentless positivity	3.1. Moving forward 3.2 Staying the helper 3.3 Positivity and people with aphasia	*We definitely work to try to, you know, keep her spirits uplifted…* *It's kind of have having to stay up and positive and meeting their needs, I guess, um, being motivating*. *… he hated me being a cheerleader… He just feels like it should have been better. He should be able to do more*.
4. Resentment, anger, and sadness		*… you can't just not try… that would infuriate me… You don't get to say that you're hopeless*.

### 3.1 Shapeshifting to Manage Many Roles

Care partners described a drastic shift in their sense of self as they took on multiple new roles and responsibilities to care for the person with aphasia. This superordinate theme captures how caregiving shifted their identities socially, emotionally and economically. Three distinct yet overlapping subthemes also emerged, reflecting roles that extend beyond physical responsibilities and shape the way care partners perceive themselves within the family.

#### 3.1.1 Beyond the Sole Provider

When people with aphasia experience changes in employment following their stroke, the career trajectories and financial situations of care partners are often upended abruptly. In addition to being the main source of income, many care partners also become the sole financial managers in their homes.

Angela, whose husband managed the finances prior to his aphasia, reflected on her experiences:

‘He was… not only the breadwinner really of the family… he was also… the person who did all of the finances… all the bills… I learned as we went.’ [Angela, spouse]

Angela's description highlights both the practical and emotional labour associated with caregiving. The comment ‘learned as we went’ suggests the evolving nature of her care partner experience as she took on new responsibilities. She had to manage the finances and accept the new care partner identity as she progressed in her caregiving journey.

Rose, another spouse, echoed these sentiments, and shared how she had to change her career entirely:

‘I'm with you… [husband with aphasia] did everything. He was… chief breadwinner, he was, he did all of the planning, all of that. So, that was a really hard turn for me, too. I actually switched careers. I'm still in the school… I needed to make more money, right?’ [Rose, spouse]

Like Angela, Rose had to take on the responsibility of managing finances for her family. She also changed careers because of these additional financial burdens resulting from her spouse's aphasia. The career change was ‘really hard’ for Rose as she was forced into this role after her husband's stroke. Both women used the term ‘breadwinner’ to describe their husbands’ previous roles, yet did not apply this term to themselves. It's possible that traditional gender role expectations may influence identity construction without being fully internalized. All participants agreed that the abrupt shift in financial responsibilities placed significant burden on them and altered their sense of self and family dynamics.

#### 3.1.2 An Informal Therapist

Care partners also expressed having to take on the role of ‘informal therapist' to help people with aphasia in their rehabilitation process. This new role involves persistence and creativity but can also take an emotional toll on the care partners.

Angela described turning daily activities into therapy sessions for her husband:

‘I've, like, tied his right hand, like, to his side, so that he has to use his left hand. I ask him questions like, I know exactly what he would like me to cook for dinner, and I make him like, try to explain to me all the different details and what goes into it.’ [Angela, spouse]

Angela took on the roles and responsibilities of a health professional to adapt daily life activities into rehabilitation exercises for her husband. This presents yet another way care partners step into new identities in the recovery process, balancing more obligations in the home while simultaneously fostering the development of their partners’ skill sets. Angela continued to engage her husband in rehabilitation, as insurance was no longer covering his therapy sessions.

Care partners also talked about some of the creative strategies they use at home in the absence of formal therapy. Alicia, caring for her mother, described a strategy which she uses during casual conversations:

‘My mom was like, ‘Hey, did you watch this show?’ I'm like ok, tell me about it. Tell me what the synopsis is… even if I've seen it already, just to get her to tell me what it is…’ [Alicia, adult daughter]

Despite having seen the show already, Alicia asked for the synopsis of the TV show as an intentional therapeutic exercise designed to engage her mother. Alicia's experience with her mother is an example of how care partners may find creative ways to engage their family members with aphasia, often using mutually enjoyable activities to implement therapeutic techniques. The therapist role places more emphasis on enriching the person with aphasia's communication rather than mutual exchanges, which is a shift from Alicia's previous experiences in her mother‐daughter relationship. These subtle shifts in relationship dynamics could have downstream consequences on emotional intimacy as roles and identities within the relationship changed.

#### 3.1.3 A Social Middleman

Many care partners become social mediators, advocating for the person with aphasia and buffering their social interactions to prevent misunderstandings or hurt feelings. Jonathan, a spouse, reflected on how others misunderstand the permanence of aphasia:

‘… they just don't get that aphasia isn't something that you just recover from, and… that does make it tough.’ [Jonathan, spouse]

The general lack of understanding of aphasia can make it challenging for people with aphasia to maintain some of their relationships, and care partners often take on the responsibility of explaining the chronicity of aphasia to others. This is an important aspect of care partner burden because care partners shape‐shift into advocates for their family members, shielding their family members from difficult conversations surrounding the nature of their aphasia.

Care partners may also intervene in the relationships of people with aphasia to prevent miscommunication or conflict with others. Michelle, the sister of a person with aphasia, shared,

‘I had to explain to family members… if [person with aphasia] calls you at 11 o’ clock at night… he doesn't get that like, that you have to go to work…please don't get upset with him… you know, because he's just trying. He just wants to talk to somebody…’ [Michelle, sister]

Michelle's role as peacekeeper involved pre‐emptively managing miscommunications that could damage family relationships. She served as a relational buffer, ensuring that her brother with aphasia would not be misunderstood or rejected. The clarification that her brother ‘just wants to talk to somebody’ highlights the advocacy role that care partners often take on. Assuming an advocacy role can be emotionally taxing as care partners feel responsible for mediating social situations to prevent misunderstandings, but it can also be meaningful as they serve as a bridge between their family members with aphasia and the broader community.

Across focus groups, care partners described how shifting roles (e.g. provider, informal therapist, advocate) reshapes their sense of self and alters how they perceive their connections with the person with aphasia, frequently resulting in changes in both family and romantic intimacy.

### 3.2 Loss of Intimacy

Aphasia's impact on care partners extends beyond their individual identity; it can disrupt intimacy in marriage, family, and friendship, forcing a redefinition and fracturing the shared identity they have with the person with aphasia they care for. Ten out of thirteen care partners discussed the surface‐level nature of their conversations with the person with aphasia which altered their emotional intimacy.

#### 3.2.1 Communication Without Depth

Care partners discussed how aphasia can interfere with having meaningful conversations with their loved ones, altering the level of depth their conversations reach compared to before the onset of aphasia. Edna, the spouse of someone with aphasia, powerfully articulated this:

‘… the thing with aphasia is he changed so much, like, I'm really [getting] to feel that I know he, the new person that he became because there's no depth, you know, there's… communication, but no depth…’ [Edna, spouse]

Edna's reflection conveyed a disruption in intimacy. While she still converses with her husband, she experiences their conversations as less layered than before. She described her husband as a ‘new person’, suggesting that aphasia changed the way he communicates and how she relates to him. The phrase, ‘…communication, but no depth’ reflects her frustration with how effortful and limited their conversations can feel since her husband's aphasia. These statements by Edna also point to her experience of ambiguous loss (i.e. someone being physically present but psychologically absent). For Edna, her husband remains with her physically, but the ease and intimacy that characterized their pre‐aphasia conversations now feel altered as she adjusts to ‘the new person that he became.’

#### 3.2.2 Pretending in Conversations

Some care partners discussed concealing their true feelings in conversations to protect the person with aphasia's confidence. This protective buffering, in turn, can impact the emotional intimacy present in different conversations. Michelle, the sister of someone with aphasia, said,

‘You know, I'm not knowin’ if it was a yes question or a no question or what I'm supposed to respond. But it was like, Oh, okay, okay.’ [Michelle, sister]

Michelle described how she might pretend to understand her brother, revealing the performative labour care partners may undertake to preserve their familial harmony. While Michelle's intention was to protect her brother's well‐being, pretending to understand him can create an illusion of intimacy based on pretense rather than the genuine understanding typically found in familial relationships.

Other care partners talked about how the laborious nature of conversations with their family members with aphasia resulted in loss of intimacy as they did not want to discuss and try to understand each other for hours on end. Patricia, caring for her adult son, stated,

‘I keep saying I understand it, and then he wants it repeated word for word, which by now my mind has lost complete interest in whatever we were talking about.’ [Patricia, mother]

Patricia described conversations with her son as requiring much more patience and repetition than those she has with people who do not have aphasia, and her focus often shifts from the original topic to making sure she understands him. Care partners reported difficulty in feeling connected to the people with aphasia they care for as their conversation topics often lack depth and become repetitive. Here, Patricia feels disconnected from her son as she anticipates conversations with him to remain at a surface‐level. Communication with someone who has aphasia may feel more procedural rather than relational for care partners, causing them to disengage.

#### 3.2.3 Erosion of Intimacy

Beyond the difficulty of maintaining conversational depth with the people they care for, spousal care partners face additional challenges of preserving intimacy in their conversations and partnerships. Paul, a husband, noted,

‘The intimacy. I know I'm talking from a husband, wife, situation. You know, the int‐ intimacy definitely changed….’ [Paul, spouse]

Paul mentioned changes in intimacy in his relationship; he grieved the loss of deeper connection with his wife as he adapted to a care partner‐care recipient relationship rather than the husband‐wife relationship he is used to sharing. He mentioned this in response to a conversation about social relationships, stating the impact of his spouse's aphasia extends beyond conversation and affects the marital relationship.

Ariel, another spouse, described how the phrase ‘I love you’ became delayed or absent following her husband's stroke:

‘[Husband with aphasia], I mean, probably took 2–3 weeks to say ‘I love you’ [after the stroke]…and he still struggles, saying it, um, like we'll be going to bed at night and I'll say ‘I love you’ and he's like ‘Goodnight.’…’ [Ariel, spouse]

Ariel experienced a loss of affirmation in her marriage. The affirmation of ‘I love you’ previously conveyed a sense of shared understanding in her marriage, but that was lost immediately after the onset of her husband's aphasia. This loss became evident when Ariel's husband struggled to say ‘I love you’ and instead switched to saying ‘goodnight’, resulting in their communication feeling unnatural and stilted. Although she knew logically that his difficulty saying ‘I love you’ was due to his aphasia, she struggled to not interpret it as a sign that their emotional intimacy may be changing.

Care partners across focus groups described the loss of intimacy after the onset of their family member's aphasia (e.g. shallow conservations and disrupted expressions of love), illustrating how aphasia can alter the emotional landscape of relationships and change the way care partners experience emotional closeness with someone who has impaired language.

### 3.3 Relentless Positivity

Despite a change in intimacy, care partners described adopting a persona of unrelenting positivity, even at the expense of expressing their own complex emotions.

#### 3.3.1 Moving Forward

One care partner described keeping a positive attitude to help her mom to move forward with aphasia:

‘We definitely work to try to, you know, keep her spirits uplifted, and, you know, to keep reminding her that, you know, she's still the same person, just, you know, slightly different…unhappiness comes with [aphasia], depression comes with [aphasia]. It's how determined are you to, you know, keep pushing.’ [Alicia, adult daughter]

According to Alicia, keeping her mom's ‘spirits uplifted’ is a critical aspect of helping her to move forward with a new sense of normalcy. Although it requires effort to ‘push’ her mom to overcome her hardships, Alicia is focused on helping her mother move forward and exist with a more positive state of mind. Providing positive reinforcement is both a dimension of care in the recovery process for people with aphasia and an implicit aspect of identity that care partners develop.

#### 3.3.2 Staying the Helper

Several care partners shared that they put significant energy towards being motivational for their family members with aphasia and embracing their responsibilities as care partners. One care partner shared,

‘… I just try to really keep a positive… mood in me going, um, you know, hers can vary a little bit, but I like to try and just stay the caregiver, you know… Stay the helper… and try to get through whatever needs to be.’ [Jonathan, spouse]

Jonathan indicated that he refrains from sharing about his mood to his wife with aphasia by saying he tries to ‘stay the helper.’ Though he may have more complicated feelings about caring for someone with aphasia, he prioritizes his role as the care partner and tries to stay positive for his spouse. Identifying as ‘the helper’ is a significant way that aphasia can impact care partners psychologically; care partners may dismiss their own emotional and physical needs in favor of the unfailing positivity they believe is needed for the person with aphasia to live successfully.

Another care partner corroborated this statement. He said,

‘I'm on the same page as that, too. It's kind of have having to stay up and positive and meeting their needs, I guess, um being motivating.’ [Hunter, spouse]

Hunter described spending significant time and energy ‘being motivating’ for his spouse with aphasia. ‘Having to stay up’ conveys his perception of positivity as a constant state that must be upheld, a stance that may contribute to the suppression of his own deeper emotions.

#### 3.3.3 Positivity and People With Aphasia

While some people with aphasia appreciate this boost in positivity, one care partner described how her positivity and motivation may be negatively received by her husband. She said,

‘… he hated me being a cheerleader, hated it. He can see his progress…when he should feel proud of his accomplishments, he doesn't… He doesn't feel like he failed. He just feels like it should have been better. He should be able to do more.’ [Ariel, spouse]

Ariel reflected on her attempt to be a ‘cheerleader’ for her husband's progress. While her intention was to help her husband see the good in his accomplishments, his focus was on his own unmet expectations and internalized sense of failure. Her husband's rejection of her positivity is an example of how people with aphasia may reject expressions of positivity when they feel contradictory to their internal experience. As demonstrated by Ariel, care partners may lean into unrelenting positivity and encouragement even when it's not wanted by the person with aphasia. This tendency may be driven by internalized expectations to remain strong and supportive, protect the emotional well‐being of their family member with aphasia, or the need to affirm their own sense of productivity as a care partner. Over time, however, this pattern of positivity may lead to relational strain between the care partner and person with aphasia.

### 3.4 Resentment, Anger and Sadness

While most care partners talked about positivity as a strategy to motivate themselves and the person with asphasia they care for, others expressed feelings such as resentment, anger, and sadness as being intrinsic to the caregiving experience.

One care partner opened up about her frustrations with her husband who has aphasia, insisting that he stay determined and hopeful throughout his rehabilitation. She said:

‘I would strangle my husband like I would hardcore like, strangle him like he would be dead, because I would be like you can't just not try… that would infuriate me… You don't get to say that you're hopeless.’ [Angela, spouse]

Angela's hyperbolic use of the word ‘strangle’ and other violent imagery reveals her feelings of anger and resentment towards her husband. She leaves no room for her husband to express hopelessness, suggesting that would ‘infuriate’ her. This quote also highlights the pressure care partners face to persist as strong and optimistic supports for the people they care for. Over time, however, maintaining that persona can lead to resentment and emotional exhaustion.

Rose, the spouse of a person with aphasia, dropped the notion of positivity and stated,

‘You know, sometimes I am angry, like, I wish I didn't have to do all this right. I hate that I have to…’ [Rose, spouse]

Rose echoed feeling resentment toward her spouse with aphasia as she talked about an experience where she didn't want to do something but felt obliged to. Her resentment suggests that caregiving is not just burdensome but can feel forced due to socio‐moral norms. As family members take on the role of care partner, they may be tasked with responsibilities they don't want to do. Bringing awareness to more negative and complex emotions, such as resentment, can create space for care partners to receive the support they need.

Alexander, the parent of a person with aphasia, stated,

‘I was in a meeting recently with five people, and two of them had left their spouses who had aphasia because they couldn't deal with it. And they cried… I personally have not had that experience. I've been puzzled many times, and I've shed a tear.’ [Alexander, parent]

Alexander talked about how care partners experience burnout and emotional exhaustion to the point where some of them leave their partners. He clarified that he hasn't personally had that experience but has ‘shed a tear’, which may point to him maintaining a facade of positivity so that he can be perceived as a resilient care partner due to societal expectations rather than admitting that he too has faced emotional exhaustion.

## 4 Discussion

The purpose of this study was to explore the subjective experiences of care partners and the ways their identities transform in aphasia caregiving, including how they navigate different dimensions (e.g. emotional, relational) of the caregiving role. Data were analysed using IPA. Four superordinate themes were developed that describe the complex and identity‐impacting dimensions of caregiving for care partners of people with aphasia: (1) *Shapeshifting to manage many roles*; (2) *Loss of intimacy*; (3) *Relentless positivity* and (4) *Resentment, anger and sadness*. Results from this study suggest that caregiving for someone with aphasia is an ongoing process that involves practical, emotional, relational, and identity‐related dimensions that shape the lived experiences of care partners.

Participants reported experiencing dramatic shifts in their daily routines, responsibilities and relationships, which pushed them to accept new identities where they had to provide both practical and emotional support to their family members with aphasia. Many people with aphasia are unable to return to work following stroke, which places a significant financial burden on care partners due to loss of income and increased cost of care (Jacobs and Ellis 2023). This was reflected in our study, as care partners indicated they had become the sole providers. Furthermore, female spouses in this study often described their husbands as ‘breadwinners’ but did not use this term for themselves. It's possible that traditional gendered expectations around financial responsibility remain influential even when female spouses have taken on that role as part of their caregiving duties (Gaunt 2013). Beyond shifts in financial responsibilities, care partners in this study also discussed becoming informal therapists for the people with aphasia they care for. These results are in line with previous research showing that care partners play an ongoing role in supporting communication practice for people with aphasia across difference contexts (Winkler et al. 2014; Shafer et al. 2019). Taking on this role, however, may be burdensome for care partners as they try to turn mundane interactions into therapeutic exercises without adequate support and training. Providing therapeutic support for the person they care for may make their relationship feel more procedural rather than relational. Care partners also noted feeling responsible for helping their family members with aphasia navigate social situations due to their communication impairments, and as a result, they experience changes in their own social identity. This added responsibility places an additional burden on care partners as they juggle multiple roles to support their family member's participation and comfort in social interactions while also trying to maintain their own sense of identity. These findings are consistent with previous research demonstrating the various roles assumed by care partners and the associated burdens that accompany these responsibilities (Bakas et al. 2006; Shafer et al. 2019; Aslan and Altuntaş 2024). Care partners experience identity changes that extend beyond taking on new roles and responsibilities. They also undergo shifts in how they relate to their family members, as the aftereffects of stroke and aphasia disrupt their normal routines and alter their perceptions of these relationships (Eifert et al. 2015).

Care partners also discussed experiencing ambiguous loss (i.e. a person, who is ‘physically present, yet psychologically absent’) (Boss 2011, 2016). This phenomenon is often observed in Alzheimer's disease and other dementias because of the gradual decline in communication and relationship between the patient and care partner (Westrelin et al. 2024). However, in post‐stroke aphasia, such loss can be felt instantaneously, and care partners may not acclimate to their new relational dynamics. In this study, care partners reported feeling emotionally distant from their family members with aphasia, despite being physically near them. Indeed, one care partner lamented how getting a response to ‘I love you’ is laborious and how it negatively impacts their spousal intimacy. Additionally, non‐spousal care partners indicated how they sometimes put on an appearance of understanding to preserve their relationship with the person with aphasia they care for, despite feeling the conversation lacks depth. Results from this study highlight that even those closest to people with aphasia can struggle to communicate effectively, which ultimately impacts their sense of connection. With proper support and resources, care partners would not have to pretend in conversations with the people they care for.

Another dimension of caregiving identity that emerged in this study was the care partner's perceived need to maintain a positive attitude for the people with aphasia they care for. Care partners indicated that they try to be positive for people with aphasia and continue serving as motivators, even when it is difficult. This protective buffering (Langer et al. 2009), in which care partners try to remain positive while hiding aphasia‐related concerns, may serve as a coping strategy used to shield the people with aphasia they care for from societal pressures. Care partners described going beyond protective buffering and maintaining a relentlessly positive attitude at the expense of expressing their own emotions. This enforced optimism by care partners appears to serve multiple functions: protecting the emotional well‐being of people with aphasia, maintaining the care partner's sense of agency and purpose, and providing a framework for hope in an uncertain situation. Care partners’ resilience may be shaped not only by individual circumstance, but also by sociocultural expectations in family caregiving experiences as it relates to perceived expectations of caregiving (Pharr et al. 2014).

Relentless positivity may also contribute to tension between care partners and people with aphasia as some care partners feel compelled to conceal the emotional experiences they find painful or difficult. This suppression of emotions, as a result, may get in the way of authentic emotional exchange (Gillespie et al. 2010). We found that tension between care partners’ outward presentations and internal emotional states became a significant source of distress; the more they felt pressured to reframe their difficult emotions, the more intense their negative emotions became. In this way, relentless positivity did not buffer their negative feelings but instead amplified them, contributing to feelings of resentment, anger and sadness. Our results support existing literature, which shows that the expectation to stay positive can heighten negative feelings such as guilt, sadness, and resentment in care partners who already feel burdened and distressed because it conflicts with their lived emotional reality ((Qiuchang) Cao et al. 2026).

Our findings can be interpreted through CIT, which postulates that there are five phases to care partner identity development (Montgomery and Kosloski 2012). In Phase II, care partner identity begins when the carer realizes that carer activities are beyond familial roles, and they begin to self‐identify as care partners. In Phase III, care needs increase beyond initial familial relationships, and there is tension between maintaining one's familial identity versus care partner identity. In our study, the onset of aphasia established the caregiver identity and resulted in downstream consequences to how care partners navigated their sense of self. Moreover, the complexity of roles and changes in intimacy levels while trying to maintain a semblance of normalcy suggest that care partners’ identities oscillate between their pre‐ and post‐aphasia relationships. Care partner identity may also be fluid and flexible, often influenced by socio‐cultural contexts in addition to their unique relationship to the care recipient.

## 5 Clinical Implications

Care partners in this study discussed experiencing identity changes and shifts in relational roles, often navigating a transition from a familial role (e.g. sibling, spouse) to a role defined by caregiving responsibilities (e.g. informal therapist, financial provider). These disruptions in their relational identities were closely tied to emotional strain and loss, experiences that typically go unaddressed in traditional rehabilitation settings. Clinicians could incorporate these findings by designing family education that includes facilitated discussions or therapeutic exercises to address topics such as identity shifts and emotional complexity. Additionally, care partners could benefit not only from resources on aphasia and communication strategies but also from structured opportunities to reflect on how caregiving reshapes their sense of self and their relationships with others. Previous work has focused on spousal relationships, highlighting the need for interventions that explicitly address intimacy following aphasia (Stead and White 2019). Similar opportunities may be beneficial across a broader range of relationships, where relational changes also occur.

Care partners in this study also described feeling pressured to maintain a positive attitude, both for the sake of their family member with aphasia and to meet perceived expectations from themselves, their family or society. Given that care recipient health outcomes may be dependent on care partner health and well‐being, interventions should prioritize care partner well‐being alongside that of the care recipient (Badesha et al. 2023). Interventions that include components of psychoeducation, skill‐building, and family education that directly supports their psychosocial adjustment could play an important role in reducing care partner burden and increasing their psychological well‐being (Le Dorze and Signori 2010; Iwasaki et al. 2023; Hernandez et al. 2025). Family education should create a space for emotional honesty, validating the full range of care partner experiences rather than reinforcing a singular narrative of relentless positivity. By considering the relational, emotional, and identity‐level dimensions of caregiving, clinicians can better equip care partners to navigate the challenges of caregiving for someone with aphasia and ultimately improve well‐being for both care partners and people with aphasia.

## 6 Limitations

Several limitations must be acknowledged. First, we did not perform formal member checking. Although member checking can enhance the credibility of findings, we felt asking care partners to participate beyond the focus group may place additional burden on them and risk reducing participation altogether. Additionally, we concluded that member checking was not necessary as IPA positions the researcher as an active meaning‐maker within the double hermeneutic process. That said, we ensured our interpretations remained grounded in participants’ accounts in multiple ways, such as frequently confirming accurate understanding of participants’ accounts in real time during the focus groups, doing multiple reviews of the data, and holding numerous discussions among the research team to ensure themes represented the participants’ expressed experiences.

Additionally, it is possible that care partners wanted to present themselves positively in the focus group setting due to social desirability, which can lead to underreporting of the negative aspects of caregiving. These findings may lean toward more optimistic perspectives, potentially overlooking more challenging aspects of participants’ experiences (Bergen and Labonté 2020; Bispo Júnior 2022).

Finally, many participants in this study held graduate degrees (*n* = 7), which may reflect educational biases that limit the generalizability of the study's findings. Participant perspectives captured in this study may not fully represent individuals with different educational backgrounds.

## 7 Future Directions

Findings from this study suggest that care partners undergo profound identity shifts, necessitating support in reconciling their new sense of self with their former identity. Prior research highlights the emotional burdens that spousal care partners experience as communication impairments disrupt martial dynamics (Dar and Biran 2025; Reed and Hinckley 2025). Studies also suggest that group therapy can strengthen marital relationships in aphasia care (Rasmus and Orłowska 2020). Further work is needed to examine how these therapeutic approaches can be adapted or extended to the diverse caregiving relationships involved in aphasia care. Additionally, future studies should examine interventions that address mental health, ensuring that both care partners and individuals with aphasia benefit from the treatments and experience improvements in overall emotional well‐being. Lastly, it remains unclear how pre‐aphasia relationship dynamics shape the development of post‐aphasia care partner identities, including the extent to which individuals accept or resist the care partner role. Understanding these relational foundations is important for interpreting variations in care partner experiences and how support needs may differ across individuals.

## Funding

S.A.A. was supported by the National Institute on Deafness and Other Communication Disorders of the National Institutes of Health under award number K23DC020757.

## Ethics Statement

This study was approved by the Northwestern Institutional Review Board (STU00217329). All participants provided written consent.

## Conflicts of Interest

The authors declare no conflicts of interest.

## Supporting information



Supporting Information: jlcd70290‐supp‐0001‐SuppMat.xlsx

## Data Availability

The data are available upon request from the corresponding author.
